# Factors associated with sleep disorders among adolescent students in rural areas of China

**DOI:** 10.3389/fpubh.2023.1152151

**Published:** 2023-04-17

**Authors:** Dan-Lin Li, Xin-Yi Nie, Jun Li, Yi-Jin Tao, Chun-Hua Zhao, Hua Zhong, Chen-Wei Pan

**Affiliations:** ^1^School of Public Health, Medical College of Soochow University, Suzhou, China; ^2^Department of Ophthalmology, The Second People’s Hospital of Yunnan Province, Kunming, China; ^3^Department of Ophthalmology, The First Affiliated Hospital of Kunming Medical University, Kunming, China; ^4^Department of General Medicine, Big Data Center, The Affiliated Suzhou Hospital of Nanjing Medical University, Suzhou Municipal Hospital, Nanjing Medical University, Suzhou, China

**Keywords:** sleep pattern, sleep disorder, adolescent, rural China, student

## Abstract

**Background:**

This study aimed to determine sleep patterns and the prevalence and association factors of sleep disorders in a regionally representative sample in Mo Jiang, China.

**Methods:**

A total of 2,346 (participation rate 93.5%) Grade 7 students (aged 13–14 years) from 10 middle schools, including 1,213 (51.7%) boys and 1,133 (48.3%) girls, participated in the study. All the participants were invited to complete questionnaires that acquired information on sleep patterns, academic performance, academic stress, and sociodemographic factors. Sleep disorders were assessed using the Chinese version of the Children’s Sleep Habits Questionnaire. Logistic regression models were used to investigate factors associated with sleep disorders.

**Results:**

The prevalence of sleep disorders among rural adolescents was 76.4%, which is higher than that among urban adolescents. Compared with previous findings in urban areas, our results indicate that sleep loss is much more severe in rural adolescents. Sleep disorders were positively associated with factors, such as watching TV [odds ratio (OR) = 1.22, *p* = 0.001], academic performance (OR = 1.80, *p* < 0.001), and academic stress (OR = 1.38, *p* = 0.04). In addition, girls were more likely to suffer from sleep disorders than boys (OR = 1.36, *p* = 0.01).

**Conclusion:**

Insufficient sleep and sleep disorders have become common health problems in rural Chinese adolescents.

## Introduction

Good quality and adequate sleep plays an important role in the healthy development and optimal daytime function of adolescents (1, 2). However, sleep disorders frequently occur in adolescents. The prevalence of sleep disorders in adolescents varies from 20 to 60% in Western populations (3, 4) and is even reported to be higher in Asians (5, 6), especially in Chinese adolescents (7). Interethnic discrepancy in sleep disorders between Westerners and Asians are not completely understood. Moreover, sleep disorders in adolescents may be associated with a broad range of factors, such as sociodemographic, sociocultural, environmental, familial, and individual factors (8).

In China, a series of studies conducted in urban areas demonstrate that sleep disorders are a widespread problem in adolescents with an estimated persistence rate of 15–70% (9–11). Li et al. conducted a multicenter study on 19,299 adolescents from eight cities in China: Shanghai, Canton, Wuhan, Chengdu, Xi’an, Urumqi, Hohhot, and Harbin found that more than two-thirds of adolescents aged over 11 years have sleep disorders (12). In a study in Beijing (13), the prevalence of sleep loss (total sleep time (TST) less than 9 h/day) among 9,198 children aged 3–14 years was 11.8%. Zhou et al. reported that 34.3% of children and adolescents aged 6–14 in Shanghai have poor sleep habits (11). Of the 1,365 adolescents between the ages of 12 and 18 years old in Shandong (14), 16.9% reported symptoms of insomnia, and the prevalence of difficulty initiating sleep, difficulty maintaining sleep and early morning awakening was 10.8, 6.3, and 2.1%, respectively. In another group of 1,056 adolescents from Shandong (15), 26.2% were unsatisfied with their sleep. About 18.8% reported that their sleep quality was poor, 16.1% had insomnia and 17.9% had daytime sleepiness. In coastal cities such as Guangzhou (16), a sizeable percentage of adolescents also struggle to sleep. In a study of 912 Shenzhen (8) adolescents aged 6–14, 69.3% had sleep disorders, including bedtime resistance (22.9%) and daytime sleepiness (20.0%). In addition, 23.8% reported suffering from repeated sleep loss (TST less than 9 h/day). The students in grade 7 are under high schooling pressure and their sleep status is the most concerned problem of public health practitioners in China However, data on Chinese school students in this age, especially in rural communities, are still lacking.

China is the world’s most populous country, and populations residing in rural areas account for more than half of the country’s population.^1^ Furthermore, with the rapid development of the rural economy in China, tremendous changes in lifestyles have occurred among young generations in these areas. They are faced with a new range of time use choices and prolonged commuting times, which may encroach on sleep time, interfere with sleep patterns, and result in sleep disorders. Therefore, understanding sleep patterns and sleep disorders may have important implications from a public health perspective. This study aims to examine sleep patterns, sleep disorders and associated factors among a school-based sample of 7th graders aged 13–14 in rural China. We hypothesize that sleep disorders among rural stumaildents is common and might be associated with lifestyles and school factors.

## Methods

### Study participants

The present findings were based on data collected from the Mojiang Myopia Progression Study on students of Grade 7 from 10 secondary schools, which was conducted in 2016 (17). Mojiang is a small rural county located in Southwestern China with a population of 0.36 million and an area of 5,312 km^2^. It was chosen as the study site due to its relatively stable demographic structure and similar socioeconomic status to the average of rural China. An explanatory letter about the nature of the study was sent out, and a consent form for taking full measurements was obtained from at least one parent or legal guardian of each participant. Telephone calls or home visits were made when parents could not be contacted. Ultimately, a total of 2,346 (participation rate 93.5%) Grade 7 students including 1,213 (51.7%) boys and 1,133 (48.3%) girls participated in the study. More details of the project were described in previous publications (17, 18).

Our study adhered to the tenets of the Declaration of Helsinki for research involving human subjects and was approved by the Institutional Review Board of Kunming Medical University.

### Chinese version of the children’s sleep habits questionnaire

The Chinese version of the Children’s Sleep Habits Questionnaire (CSHQ) has been used in extensive research, both in children and adolescents (19–21). The questionnaire about adolescents’ sleep habits in a typical recent week consists of 33 items grouped into eight subscales: bedtime resistance, sleep onset delay, sleep duration, sleep anxiety, night waking, parasomnias, sleep-disordered breathing, and daytime sleepiness. Each item was scored on a three-point Likert scale: 1 (“rarely, 0–1 time in a week”), 2 (“sometimes, 2–4 times in a week”), and 3 (“usually, 5–7 times in a week”). The adequate internal consistency of the overall questionnaire ranged from 0.68 to 0.78, and the test–retest reliability ranged from 0.62 to 0.79. Sleep disorders were diagnosed by a total CSHQ score of 41 points or more (19). In addition, previous studies suggested a subscale score > standard deviation (SD) above the mean score of a community sample to identify specific sleep disorders of clinical significance (19).

### Sleep-related variables and demographics

A predesigned questionnaire was used to collect information regarding demographic characteristics that were potentially associated with sleep disorders, such as academic performance, time spent on TV, time spent surfing the internet, time spent outdoors, family structure, and parental education level. With the help of parent (s), the adolescents completed the questionnaire at home for 2 days. After completion of the questionnaires, the research assistants checked the questionnaires to ensure that all questions were properly answered.

Academic performance was determined *via* self-report measures. The adolescents responded to the question “how is your academic performance?” on a three-point Likert scale from 1 (“excellent”), 2 (“moderate”), and 3 (“underachievement”). Adolescents filled out their usual bedtime and wake time on weekdays and weekends, and the research assistants calculated the total sleep duration.

### Statistical analysis

Sleep patterns, including morning wake time, bedtime, and total sleep time (TST) on weekends and weekdays, were described with means and SD. We calculated the prevalence rate of sleep disorders (total score of 41 points or more) and the number and ratio of individuals who exceeded cut-off points. Logistic regression models were used to explore the associations of sleep disorders with potential factors. First, we examined variables of interest such as lifestyle-related factors and school factors in univariate models. Second, we included sex and factors with a *p* value of less than 0.10 in univariate analyses in the multivariate analysis models. Odds ratios (ORs) and 95% confidence intervals (CIs) were calculated to quantify the associations. Statistical tests were set with a significance level of 0.05. Statistical Package for Social Science version 20.0 was used for all the analyses.

## Results

Descriptions of demographic characteristics, media (computer and TV), family structure, and academic performance are shown in Table 1. The mean age of the adolescents was 13.6 (SD: 0.5) years old, and among the total number of adolescents, girls accounted for 48.3% (1,133). Most of their parents graduated from junior high school or below, with only 17.0% (399) of their fathers and 12.4% (270) of their mothers who graduated from college or had a higher degree. Regarding school factors, 28.4% of adolescents underestimated their academic achievement and 89.1% of the students thought they experienced academic pressure.

**Table 1:** Distributions of children by their characteristics.

	*N* = 2,346
Age in years, mean (SD)	13.6 (0.5)
Girls, no. (%)	1,133 (48.3)
Compute Use (yes), no. (%)	323 (13.8)
Time on computer per day, hours (SD)	0.9(0.9)
Time on watching TV per day, hours (SD)	1.4(0.9)
Time outdoors per day, hours (SD)	1.3(1.0)
*Father’s Education, no. (%)*	
Informal education	84 (3.6)
Primary school	760 (32.4)
Junior high school	1,029 (43.9)
High school	292 (12.4)
College school or above	107 (4.6)
*Mother’s education, no. (%)*	
Informal education	253 (10.8)
Primary school	982 (41.9)
Junior high school	717 (30.6)
High school	222 (9.5)
College school or above	68 (2.9)
*Family structure, no. (%)*	
Nuclear family	743 (31.7)
Extended family	1,504 (64.1)
Single family or others	99 (4.2)
*Academic performance, no. (%)*	
Excellence	416 (17.7)
Moderate	1,195 (50.9)
Underachievement	667 (28.4)
Academic stress (yes), no. (%)	2090 (89.1)

SD, standard deviation.

**Figure 1:**
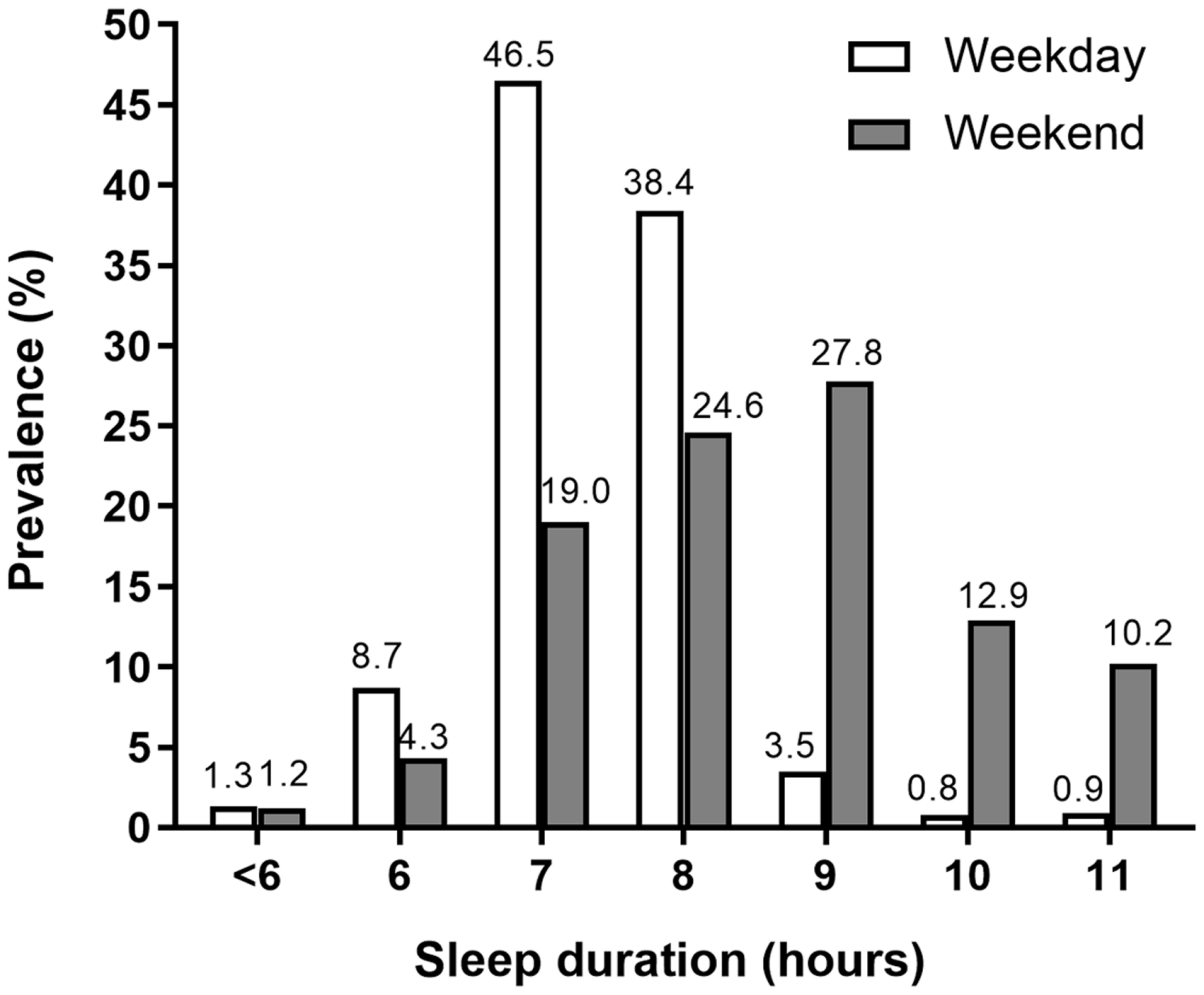
Distribution of weekday and weekend total sleep time (TST) in 2,346 Chinese adolescents aged 13–14 years.

Their sleep patterns during weekdays and weekends and the distribution of TST are shown in Table 2 and Figure 1, respectively. In our study, the adolescents woke up much earlier on weekdays than on weekends, but their bedtime was similar, accounting for significantly longer weekend TST than weekday TST. Figure 1 shows that 27.8% of the adolescents could sleep 9 h on weekends, while during weekdays 46.5% of the adolescents could sleep 7 h, and 10% of the adolescents could only sleep 6 h or less. Compared to urban adolescents, rural adolescents go to bed later, which results in an average loss of one-hour sleeping time.

**Table 2:** CSHQ score and sleep pattern.

	Rural			Urban^a^
	Mean (SD)	Cut-off	Score > cut-off (%)	
*Sleep patterns (Weekday)*
Morning wake-up time, h	6.5 (0.5)			6.0 (0.5)
Bedtime, h	22.4 (1.1)			21.4 (0.8)
Total sleep time, h	8.0 (1.2)	8.0	56.5	9.0 (0.8)
*Sleep patterns (Weekend)*
Morning wake-up time, h	7.8 (1.4)			8.2 (1.0)
Bedtime, h	22.5 (0.9)			22.2 (0.9)
Total sleep time, h	9.3 (1.5)	8.0	24.5	9.8 (1.1)
*CSHQ subscales*
Bedtime resistance	9.0 (1.4)	10.8	9.7	33.1
Sleep onset delay	1.9 (0.8)	2.3	28.6	25.4
Sleep duration	5.4 (1.5)	5.3	50.3	70.1
Sleep anxiety	4.8 (1.3)	7.8	5.9	41.4
Nighttime awakening	3.5 (0.9)	5.3	3.7	27.4
Parasomnias	8.3 (1.7)	10.6	10.4	52.4
Sleep disordered breathing	3.4 (0.8)	4.5	9.2	11.9
Daytime sleepiness	12.5 (3.6)	15.2	23.5	71.2
Total CSHQ score	48.9 (8.0)	41.0	76.4	

CSHQ, children’s sleep habits questionnaire.

SD, standard deviation.

a The study sample was recruited from eight urban cities in China, including Urumqi, Chengdu, Xi’an, Hohhot, Wuhan, Canton, Shanghai, and Harbin, aged 11 years (12).

The total CSHQ and subscale scores are listed in Tables 2, 3, respectively. Approximately 76.4% of adolescents with a CSHQ total score above 41 suffer from sleep disorders, and sleep duration (50.3%) is the most prevalent sleep disorder among the eight specific sleep disorders. Based on the CSHQ score, 72.6% of adolescents reported that they *“*fall asleep in their own bed*”* 5–7 times a week, which belongs to the bedtime resistance subscale. Except for sleep onset delay, other specific sleep disorders were reported more frequently by the urban adolescents, as shown in Table 3.

**Table 3:** The prevalence of specific sleep disorders.

CSHQ subscales	Usually, No. (%)	Sometimes, No. (%)	Rarely, No. (%)
*Bedtime resistance*
Going to bed at same times	726 (30.9)	977 (41.6)	643 (27.4)
Falling asleep in own bed	1,703 (72.6)	254 (10.8)	389 (16.6)
Falling asleep in other’s bed	53 (2.3)	166 (7.1)	2,127 (90.7)
Needing parent in room to sleep	17 (0.7)	112 (4.8)	2,217 (94.5)
Struggling at bedtime	17 (0.7)	79 (3.4)	2,250 (95.9)
Afraid of sleeping alone	95 (4.0)	248 (10.6)	2,003 (85.4)
*Sleep onset delay*
Falling asleep in 20 min	671 (28.6)	824 (35.1)	851 (36.3)
Sleep duration			
Sleeping too little	362 (15.4)	759 (32.4)	1,225 (52.2)
Sleeping the right amount	877 (37.4)	726 (30.9)	743 (31.7)
Sleeping same amount each day	485 (20.7)	779 (33.2)	1,082 (46.1)
*Sleep Anxiety*
Needing parent in room to sleep	17 (0.7)	112 (4.8)	2,217 (94.5)
Afraid of sleeping in the dark	131 (5.6)	351 (15.0)	1,864 (79.5)
Afraid of sleeping alone	95 (4.0)	248 (10.6)	2,003 (85.4)
Trouble sleeping away	164 (7.0)	457 (19.5)	1,725 (73.5)
*Night awakening*
Moving to other’s bed in night	16 (0.7)	73 (3.1)	2,257 (96.2)
Awakening once during night	126 (5.4)	596 (25.4)	1,624 (69.2)
Awakening more than once	52 (2.2)	225 (9.6)	2,069 (88.2)
*Parasomnias*
Wetting the bed at night	8 (0.3)	67 (2.9)	2,271 (96.8)
Talking during sleep	54 (2.3)	347 (14.8)	1,945 (82.9)
Restless and moves a lot	170 (7.2)	434 (18.5)	1,742 (74.3)
Sleepwalking	23 (1.0)	65 (2.8)	2,258 (96.2)
Grinding teeth during sleep	44 (1.9)	136 (5.8)	2,166 (92.3)
Awakening screaming, sweating	36 (1.5)	297 (12.7)	2,013 (85.8)
Alarmed by nightmare	148 (6.3)	647 (27.6)	1,551 (66.1)
*Sleep disordered breathing*
Snoring loudly	25 (1.1)	191 (8.1)	2,130 (90.8)
Stopping breathing	28 (1.2)	150 (6.4)	2,168 (92.4)
Snorting and gasping	55 (2.3)	288 (12.3)	2,003 (85.4)
*Daytime sleepiness*
Awakening by himself	715 (30.5)	523 (22.5)	1,102 (47.0)
Awakening up in negative mood	241 (10.3)	471 (20.1)	1,634 (69.7)
Awakening by other’s	318 (13.6)	480 (20.5)	1,548 (66.0)
Hard time getting out of bed	448 (19.1)	542 (23.1)	1,356 (57.8)
Takes long time to be alert	220 (9.4)	444 (18.9)	1,682 (71.7)
Seems tired during the day	206 (8.8)	659 (28.1)	1,481 (63.1)
Asleep while watching TV	734 (31.3)	559 (23.8)	1,053 (44.9)
Asleep while riding in car	335 (14.3)	528 (22.5)	1,483 (63.2)

CSHQ, children’s sleep habits questionnaire.

Logistic regression analyses were used to determine the factors associated with sleep disorders, and the results are shown in Table 4. Univariate analyses revealed that sleep disorders were significantly associated with gender, time spent on watching TV, family structure, academic performance, and academic stress (all p < 0.05). In the multivariate analyses, time spent watching TV (OR: 1.22; 95% CI: 1.08–1.37) and academic stress (OR: 1.38; 95% CI: 1.03–1.91) were positively associated with sleep disorders. Girls were 1.36 (95% CI: 1.31, 2.49: 1.10, 1.68) times more likely to have sleep disorders than boys. Furthermore, sleep disorders are positively associated with adolescents’ academic performance in school. After controlling for gender, academic stress, time spent watching TV, and family structure, adolescents with average academic performance were 1.32 (95% CI: 1.06, 1.67) times and underachieving adolescents were 1.80 (95% CI: 1.31, 2.49) times more likely to have sleep disorders compared with academically excellent adolescents.

**Table 4:** Logistic regression model of factors associated with sleep disorders (*N* = 2,346).

Variables	Univariate logistic regression		Multiple logistic regression	
	OR (95%CI)	*p*	OR (95%CI)	*p*
Sex (Girls vs. Boys)	1.27 (1.04, 1.55)	0.02	1.36(1.10, 1.68)	0.01
Ethnicity (Han vs. Monitory)	1.09 (0.84, 1.42)	0.25	-	
Time spent on TV, per hour increase	1.22 (1.08, 1.36)	0.001	1.22(1.08, 1.37)	0.001
Time spent outdoors, per hour increase	0.95 (0.84, 1.06)	0.34	-	
Computer use (yes)	1.05 (0.62, 1.15)	0.28	-	
*Father’s education (category)*
Informal education	**Reference**			
Primary school	1.28 (0.70, 2.33)	0.43		
Junior high school	1.35 (0.75, 2.45)	0.32		
High school	1.21 (0.64, 2.31)	0.55		
College school or above	1.27 (0.61, 2.67)	0.53		
*Mother’s education (category)*
Informal education	**Reference**			
Primary school	1.07 (0.76, 1.51)	0.71		
Junior high school	1.00 (0.70, 1.43)	0.99		
High school	1.05 (0.67, 1.65)	0.82		
College school or above	1.54 (0.83, 2.83)	0.17		
*Family structure*
Nuclear family	**Reference**		**Reference**	
Single family or others	1.32 (1.07, 1.63)	0.01	1.23(0.65, 1.53)	0.06
Extended family	1.14 (0.69, 1.88)	0.42	1.34(0.78, 2.28)	0.28
*Academic performance*
Excellence	**Reference**		**Reference**	
Moderate	1.32 (1.06, 1.66)	0.02	1.32 (1.06, 1.67)	0.02
Underachievement	1.70 (1.24, 2.33)	0.001	1.80 (1.31, 2.49)	< 0.001
Academic stress (yes)	1.37 (1.01, 1.85)	0.04	1.38 (1.03, 1.91)	0.04

OR, odds ratio; CI, confidence interval.

## Discussion

The prevalence rates reported in the present study are much higher than those reported from cities in China. In the present sample, the prevalence of sleep disorders was 76.4%. A total of 94.9% of rural adolescents had insufficient sleep (TST less than 9 h/day) on weeknights, whereas the percentage decreased to 49.1% on weekends. Moreover, a number of positive factors of sleep disorders, such as over 2 h of watching TV, poor academic performance, and academic stress, occur among rural adolescents. For rural adolescents, watching TV less than 1 h on school days was associated with lower odds of sleep disorders.

Compared with previous findings in urban areas (8, 16), our results indicate that sleep loss is much more severe in rural adolescents. In our study, the prevalence of sleep loss [total sleep time (TST) less than 9 h/day] was 94.9%. About 56.5% of the adolescents reported that they sleep less than 8 h/day, and 10.0% only sleep 6 h/day or less. The discrepancy in sleep patterns in rural adolescents may be attributed to their bedtime being much later for night lessons, which is a typical practice in rural Chinese schools (adolescents have to study in school during weeknights). The percentage of sleep loss decreases to 49.1% on weekends, which supports this notion. Given that the rural Chinese school system is different from that of urban schools, research on sleep schedules between rural and urban areas separately provides great insights into adolescents’ sleep patterns. Sleep loss in rural adolescents requires far more attention from the government and education system.

In the present study, the prevalence of sleep disorders was 78.8% in rural Chinese adolescents. Sleep duration (50.3%) was the most prevalent among the eight specific sleep disorders. These findings may partly explain why the CSHQ subscales include items that are related to strict school schedules (e.g., “going to bed at the same time,” “falls asleep in own bed,” and “sleeping the right amount”). Furthermore, the prevalence of sleep onset delay and daytime sleepiness in this study is considerably higher (e.g., 28.6% for sleep onset delay and 23.5% for daytime sleepiness) compared with a previous study conducted in eight Chinese cities on adolescents aged over 11 (12). Other reasons may also explain these findings. First, sleep is strongly shaped and interpreted by cultural values, ideological beliefs and parenting values. In China, especially in rural areas, adolescents are highly influenced by their parents and teachers. The notion of ‘knowledge changes destiny’ is widespread in rural China, and rural adolescents face great academic pressure to enter a highly regarded school. Second, the cut-offs used in this study were calculated from American children aged 4–12 (19). As the current sample is composed of Chinese adolescents and their ages extend beyond 12 years old, the prevalence determined based on these cut-offs may cause bias. Third, cosleeping (room sharing) in school is a popular practice in rural China. Positive correlations between sleep quality and cosleeping among school-aged students have been identified in previous studies (22–24).

Our study supports previous findings on urban adolescents showing that watching TV is associated with increased odds of sleep disorders in adolescents (25–27). These studies report that adolescents who watch TV more than 2 h a day experience more daytime sleepiness and problematic sleep. Another study also shows that each hour spent watching TV reduces sleep duration by 7 min (28). Although most studies cannot identify a causal relationship between watching TV and sleep disorders, sleeping patterns of urban adolescents can be influenced by watching TV. In our study, decreasing TV watching by 1 h on weekdays alleviated sleep disorders in rural adolescents. One hypothesis is that appropriate time spent watching TV may relieve rural adolescents from intensive learning tasks. Academic load and stress are risk factors for sleep disorders in adolescents (29). Further research should explore in more detail how much time spent watching TV is associated with sleep disorders and how much time spent watching TV may be beneficial for adolescents. Furthermore, no significant differences in terms of computer use with sleep disorders were reported. A possible explanation is that almost all families have TV, but computers were rarely available (13.8%) in this community.

In our study, academic underachievement was a common and serious problem that affected 28.4% of rural adolescents. Consistent with previous studies (30, 31), our study found a positive link between sleep and academic performance. In addition, 89.1% of rural adolescents suffer from tremendous academic stress. We assume that underachieving adolescents may suffer more from academic stress and tend to spend more time studying at the expense of their sleep. We further found that academic stress is positively related to sleep disorders, which supports our hypothesis. In addition, girls are more likely to suffer from sleep disorders, which is in line with a previous study in Anhui (32) and Taiwan (33) in China. Girls experiencing the menstrual period undergo fluctuating levels of estrogen, progesterone, melatonin, and cortisol, leaving them susceptible to negative effects, which may lead to sleep disorders (34). Parental educational level and family structure are not related to sleep disorders. This finding is inconsistent with the study by BaHammam et al. (35). They found that mothers’ educational level is related to adolescents’ bedtime. Another study from Maha et al. (36) reported that adolescents with a high CSHQ score had fathers with low educational levels. The effects of parental educational level and family structure on sleep disorders in adolescents warrant further investigation.

Our study provides detailed information on rural adolescents’ sleep, and we hope it can aid policymakers and support targeted interventions. At the same time, we hope it can assist professional health organizations in identifying problematic sleep and helping families to promote their children’s sleep quality.

Several limitations should also be acknowledged. First, we cannot identify a causal relationship between sleep disorders and potential factors through the cross-sectional design. Second, all measures relied on parent-reported questionnaires rather than objective assessments, which may have led to report bias. Third, although the Chinese version of the CSHQ has adequate internal consistency and test–retest reliability, the exact relationship of the parent-reported sleep disorder variables and results from objective assessment is still uncertain. Last, factors, such as seasonal effects and sleep environments, which may also affect sleep patterns and sleep disorders, should be considered in future studies.

## Conclusion

To summarize, the sample population used in the present study had an extremely higher prevalence rate of sleep disorders and sleep loss than urban adolescents. Governments and health policy makers should be aware of this issue and adopt appropriate strategies to improve rural adolescents’ sleep quality.

## Data availability statement

The datasets presented in this article are not readily available because privacy policy. Requests to access the datasets should be directed to pcwonly@gmail.com.

## Ethics statement

The studies involving human participants were reviewed and approved by the study adhered to the tenets of the Declaration of Helsinki for research involving human subjects and was approved by the Institutional Review Board of Kunming Medical University. Written informed consent to participate in this study was provided by the participants’ legal guardian/next of kin.

## Author contributions

C-WP, HZ, and C-HZ concepted the study. D-LL conducted the statistical analyses and wrote the original manuscript. JL, C-WP, and HZ participated the investigation of the study. X-YN, JL, Y-JT, HZ, C-HZ, and C-WP edited the manuscript. JL got the funding acquisition. C-WP and HZ supervised the study. All authors contributed to the article and approved the submitted version.

## Funding

The research was funded by the National Natural Science Foundation of China (grant no. 81560169).

## Conflict of interest

The authors declare that the research was conducted in the absence of any commercial or financial relationships that could be construed as a potential conflict of interest.

## Publisher’s note

All claims expressed in this article are solely those of the authors and do not necessarily represent those of their affiliated organizations, or those of the publisher, the editors and the reviewers. Any product that may be evaluated in this article, or claim that may be made by its manufacturer, is not guaranteed or endorsed by the publisher.

## Footnotes

^1^ http://www.stats.gov.cn/ztjc/zdtjgz/zgrkpc/dlcrkpc/
